# Nutrition Knowledge Translation Performance in Health Professionals: Findings from the 2017 Unified Forces Preventive Nutrition Conference (UFPN)

**DOI:** 10.3390/nu11020390

**Published:** 2019-02-13

**Authors:** Vered Kaufman-Shriqui, Hagit Salem, Ruth Birk, Mona Boaz

**Affiliations:** Department of Nutritional Sciences, School of Health Sciences, Ariel University, Ariel 40700, Israel; veredks@ariel.ac.il (V.K.-S.); hagitsa@ariel.ac.il (H.S.); ruthb@ariel.ac.il (R.B.)

**Keywords:** dietitians, knowledge translation, nutrition policy

## Abstract

Background: Dietitians and other health care professionals must be able to translate findings from clinical trials into best treatment practices, a skill termed “knowledge translation”. This skill requires knowledge of treatment guidelines as well as the science underpinning treatment recommendations. Unsatisfactory knowledge translation of medical nutrition therapy (MNT) has been documented. Methods: Individuals registered to attend a leading national nutrition conference were asked to participate in an online cross-sectional survey. Participants were asked to provide demographic and professional information, answer questions on nutrition knowledge and to choose a clinical action plan in response to dietitian-designed case vignettes describing research outcomes. Responses were compared by profession and participation in research activities. Results: Of 3000 registered conference attendees, 299 individuals replied: 79.0% dietitians, 93.3% female, with a mean household income matching the 5th decile of income, 60.7% indicated a single employment setting, 20.7% reported participating in research. Almost 74% of respondents indicated that they would make clinical recommendations based on findings of an in vitro study. In one vignette, a patient with a disease previously not encountered by the respondent required a clinical treatment plan. Only 53% of participants chose to seek formal nutrition guidelines. Fewer than 15% of participants could identify the pathway for fat during weight loss. Differences in knowledge translation skills by research participation were not detected. Conclusions: Our findings reveal a deficit in knowledge translation proficiency in a convenience sample of dietitians and other health professionals, highlighting the need to develop these skills.

## 1. Introduction

Knowledge translation (KT) is “the process of applying laboratory research to human studies and enhancing the adoption of evidence-based practices in real-world settings to reach broad populations material and methods” [1]. In 2010, The National Institutes of Health (NIH) distinguished between two areas of translation: (1) from basic science to clinical trials; (2) the translation from clinical trials to best treatment practices [2]. To occur successfully, KT requires the necessary antecedents including a distilled, integrated source of knowledge. This knowledge is derived from high-quality primary research (knowledge inquiry) [3], meaning the clinician must possess a comprehension of basic scientific underpinnings relevant to the question at hand. Translational research provides the data underlying evidence-based clinical practice and population-based health promotion efforts, thereby offering direct relevance for health professionals [4]. 

Health professionals, in a variety of fields, have not consistently conducted translational research [3,4,5,6]. In the area of nutrition, for example, poor knowledge of medical nutrition therapy (MNT) has been reported among various medical units and teams, both in the USA and Europe [6,7,8]. Factors associated with failure to conduct successful nutritional translational research were lack of knowledge and resources [6,7,8,9].

The Israeli healthcare system provides universal health coverage and is a sustainable mechanism for monitoring the quality of primary care, including the National Program for Quality Indicators in Community Healthcare (QICH) [10]. The little research that exists suggests that knowledge of MNT among Israel physicians and nurses is inadequate [11], despite agreement among both health professionals and medical students that dietary treatment and nutrition education are important [12]. 

The objectives of the present study were (1) to assess KT skills, including both source knowledge and clinical practice, among dietitians and other health care professionals participating in a large, national Israeli nutrition conference and (2) to estimate associations between sociodemographic characteristics, self-reported participation in research and KT. 

## 2. Methods

### 2.1. Overall Study Design and Plan

This cross-sectional survey was conducted in a convenient sample of nutrition conference attendees. The survey queried demographics, attitudes towards nutrition policy and KT skills. Investigators sent an online survey to all dietitians and other health professionals registered to participate in a large, national nutrition conference. 

### 2.2. Informed Consent

The present study was approved by the Institutional Ethics Committee (Helsinki Committee), Ariel University. A response to the survey indicated informed consent as stated on the first screen of the online study.

### 2.3. Study Population 

All individuals who registered to attend the 16th Annual Preventive Nutrition—Combined Forces Israel Nutrition Week Conference received a link to an online survey administered via Google Surveys. Conference organizers indicated that approximately 3000 individuals registered for the event. As this is the largest annual nutrition conference in Israel, attended by dietitians, researchers, physicians, nurses and other health professionals, the investigators selected this venue as a framework in which to query professionals in these fields. Permission was granted by the conference organizers to utilize the registration lists as a target for the population inclusion criteria

All individuals who were registered for the conference and responded to our email were sent a link to the online survey. The ability to respond to the survey, which was conducted in Hebrew, de facto included only individuals who are literate in Hebrew.

### 2.4. Facilitating Participation

To encourage participation in the survey, respondents were entered into a lottery for professionally meaningful prizes, including a digital scale and professional literature.

### 2.5. Survey Development

The survey included 36 questions, which queried demographic characteristics (age, sex, highest level academic degree, number of individuals in the household, marital status); health behaviors (exercise, smoking, dietary pattern); professional experience (profession, years in the profession, employment location, number of positions held). All demographic questions were multiple choice. The survey also included two multiple-choice questions about a recently-passed nutrition labeling law (one knowledge, one attitude) and three multiple-choice questions on nutrigenomics (knowledge), which are not included in this analysis.

A screening question asked about the respondents’ current participation in research-related activities; those who answered in the affirmative were asked to characterize their typical role in research participation (principal investigator, collaborating investigator, research consultant, research assistant). Respondents were asked to indicate the number of research abstracts they read each month and, separately, the number of full-text research articles they read each month. These questions were also multiple- choice questions. 

The survey included one knowledge question on weight loss physiology, which was translated from a survey among family doctors, dietitians and athletic trainers in the UK [13].

Additionally, the survey included two vignettes describing clinical information for which the respondent was asked to indicate his/her plan of action from a fixed set of possibilities, each with four possible answers. In the second vignette, respondents could select more than one response. Each vignette had one correct response; however, the analysis described all possible response combinations when multiple responses were possible. The vignettes were designed to explore how participants translate clinical and research information into a clinical action plan. Case vignettes have been shown to provide a valid method for assessing clinician treatment decisions [14]. Composed by three registered dietitians who conduct research and are members of the senior faculty in the Department of Nutrition Sciences, Ariel University, proposed survey vignettes were referred to a focus group of potential conference attendees to assess readability and clarity. The final version of each vignette was reviewed by registered dietitians, providing expert validity. 

### 2.6. Sample Size 

The sample size for the present study (*n* = 299), which is a convenience sample, provides 95% confidence level with a confidence interval of 5.6%. This calculation assumes a target population size of 20,000 individuals. If the target population size is limited to 3000 individuals (the number of registered conference participants), the confidence interval is 5.3%.

### 2.7. Data Analysis

SPSS v. 25.0 (IBM Inc., Armonk, NY, USA) was used for all statistical analyses. Distributions of continuous variables were approximately normally distributed as assessed using the Kolmogorov-Smirnov test; thus, these are described using mean ± standard deviation. Categorical variables such as the proportion of participants with a given response were described using frequency counts and expressed as *n* (%). Continuous variables were compared by participation in research (yes/no) using the *t*-test for independent samples, and by profession using a one-way analysis of variance (ANOVA). Associations between categorical variables were assessed using the chi-square test. All tests are two-sided and considered significant at *p* < 0.05.

## 3. Results

Table 1 describes the survey participants. The majority of participants were dietitians, female and did not describe themselves as researchers. Among the 20.7% of respondents who reported participating in research, the following roles were indicated: collaborating investigator (41.9%); principal investigator (24.2%); consultant (20.9%) and clinical research associate (12.9%). 

The areas of study for the highest degree of education included nutrition (65.4%); public health (9.2%); medical sciences (4.5%); biology (4.5%); management (3.4%); epidemiology (3.1%). Additionally, 9.9% of respondents indicated that their highest degree was in another field not specified. More than 40% of survey participants held advanced degrees.

The employment setting was not limited to a single response, so participants could indicate all settings that applied. While 60.7% of respondents indicated a single employment setting, 28.6% indicated two and 10.7% indicated three employment settings. The most frequently reported employment settings included private clinic, hospital and health maintenance organization clinic.

Categorized gross monthly household income ranged from no income to New Israeli Shekel (NIS) 33,001–58,500 (approximately $9000–16,000) per month. The mean monthly income, calculated as a weighted average of the midpoint of each category, was NIS 15,003.6 (approximately $4115.00). This compares to the 2015 Israel Central Bureau of Statistics report on monthly income and expenditure of households, which estimates the total gross money income per household at the 5th decile of income to be NIS 12961 ± 292 [15].

Table 2 presents the included knowledge question and the two vignettes included in the survey, together with the responses (%) indicated by survey participants. 

In the vignette on aspartame, the participant was told that study findings identify an association between aspartame exposure and dysregulated division in human blood cells. Though the study described was in vitro, almost 74% of participants responded that they would make clinical recommendations based on the findings, including 5.7% who would recommend refraining from aspartame consumption completely. Slightly more than one in four participants would abstain from making a clinical recommendation based on a single in vivo study. When responses were compared across professions, no significant differences in response patterns were detected. When the profession was dichotomized to a dietitian (yes/no), no significant difference in response to this vignette was observed. Further, there was no difference in response between survey respondents who did vs. did not participate in research in any capacity.

The vignette on kidney disease presented a patient with a rare and clinically complex kidney disease referred for a nutrition consultation. In this scenario, the respondent has never encountered this disease in his/her clinical experience. Respondents could select more than one answer. While the vast majority (86.6%) of respondents indicated they would seek formal treatment guidelines to guide them, only 26.4% provided this option as their only answer. The remaining participants included this response in addition to at least one other option. Almost 2/3 of respondents indicated that they would consult with a physician. Of these, only 5% provided this as their only response. Animal models would serve as a resource for 25% of respondents, but only 1% provided this as their only response. Finally, while 22% would provide the most conservative nutrition treatment they knew for patients with kidney disease, only 1.3% gave this response as their only answer. Approximately 2/3 of respondents provided multiple answers to this vignette. Two responses were given by 36.8% of respondents, of which the most frequent dual answer (26.8%) was consulting a physician and also seeking formal treatment guidelines. Another 25.1% of participants indicated three answers to this vignette, of which the most frequent combination (14.4%) included reading studies on new treatments in animal models, consulting a physician and seeking formal treatment guidelines. Four answers were provided by 4.3% of participants. 

A significant by-profession difference in response pattern was detected for responses to this vignette (*p* < 0.0001). Specifically, more dietitians (93.2%), physicians (100%) and students/interns (95.8%) indicated that they would seek formal treatment guidelines (either alone or in combination with other answers) than other professionals. The profession was dichotomized to a dietitian (yes/no). When the response to this vignette was compared by this dichotomized profession variable, more dietitians than other professionals indicated that they would seek formal treatment guidelines, whether alone or in combination with other answers: 93.2% vs. 79.7%, *p* = 0.01. Seeking formal treatment guidelines was the only answer provided by 27.7% of dietitians vs. 21.9% of other professionals, *p* = 0.35. Differences in response patterns to this vignette were not detected by participation in research.

In the knowledge question on weight loss physiology, participants were asked to identify what happens to fat when weight loss occurs. Only 14.4% of participants indicated the correct answer: fat is exhaled as CO_2_. Approximately 2/3 of respondents indicated that fat is excreted as water in urine/sweat. Identifying the correct answer did not differ significantly between dietitians and other professionals. The proportion of respondents indicating the correct answer did not differ between participants who did vs. did not participate in research.

## 4. Discussion

In this cross-sectional study that was conducted among dietitians and health professionals who participated in a leading national nutritional conference, we found that 74% of study participants chose to make a nutrition recommendation based on a single in vivo study with no difference across health professions. While 86.6% of respondents chose to seek formal nutrition guidelines in case they encountered a patient suffering from a rare disease for the first time, only 26.4% chose this option as their only response. In this instance, a significantly higher proportion of dietitians replied correctly compared to other health professionals. 

Our findings concur with previously reported results across a variety of healthcare settings and health professionals [3,4,5,6]; specifically, poor capacity to translate knowledge to clinical treatment action. 

Failure to translate research findings and best practices into clinical treatment has been shown to represent a barrier to delivering high-quality healthcare among physicians treating patients with cardiovascular diseases [16]. In Australia, clinician compliance with treatment guidelines for 22 medical conditions ranged from 32–86% in a large, nationwide, cross-sectional study [17]. A US study has estimated that <20% of physician-recommended interventions have evidence-based research to support them [18]. Inadequate MNT knowledge was documented among Scandinavian doctors and nurses; specifically, 25% of the 4512 professionals surveyed could not identify patients in need of MNT; 39% lacked techniques for recognizing malnourished patients; 53% found it difficult to calculate patient energy requirements; 66% were unfamiliar with national guidelines for clinical nutrition [7]. A British survey conducted among healthcare professionals working on intensive care units across London found that only 44% of the physicians and 26% of the nurses demonstrated an understanding of the evidence set out in the nutrition support guidelines [8]. Failure to identify social determinants of health as contributors to obesity together with lack of nutrition knowledge were among the barriers to treating adult obesity among US primary care physicians [13,19]. 

Failure to correctly recall important metabolic processes can also lead to the misapplication of clinical actions. For example, more than 60% of UK health professionals, including dietitians, failed to identify carbon dioxide and water as the end products of triglyceride oxidation, instead erroneously citing heat as the final outcome of this process [14]. In the present study, 2/3 of respondents identified water excreted in urine/sweat as the end product of fat oxidation. While complete oxidation of 10 kg of human fat requires 29 kg of inhaled oxygen producing 28 kg of CO_2_ and 11 kg of H_2_O, this is exhaled, not excreted in urine/sweat. 

Research skills sets can be improved as reported in a recent review identifying prioritization, mentoring, leadership and best practices training at all structural levels: individual, team and organizational [20]. In the present study, differences in knowledge translation skills across employment settings were not identified. This finding was not anticipated because the Israeli national health sector has developed a system of research and education for health workers that was expected to confer knowledge translation skills superior to those of private clinic practitioners [21]. On the other hand, most practitioners who work in a private clinic also work in the public health sector, thus diluting the between work setting difference. 

Nevertheless, participation in research was not consistently associated with better knowledge translation, suggesting a need to improve research capacity relevant to practice knowledge and emphasizing problem-solving to address priority health problems [22]. Educational opportunities identified as contributing to successful knowledge translation skills acquisition include interactive sessions, didactic sessions, printed material, discussion and feedback [23]. Continuing education can be provided to dietitians and other health professionals in a variety of settings including classrooms, online courses and webinars.

Illustrating this point is the 45% increase in adherence to treatment guidelines following the implementation of an educational program designed to increase knowledge translation capacity [24]. This program, conducted in an Australian weight management clinic, improved dietitian knowledge of guidelines identifying patient readiness to adopt new lifestyle behaviors. 

Findings must be understood in the context of study limitations. First, the data are cross-sectional and, as such, causality cannot be inferred. Associations between sociodemographic or professional characteristics and knowledge translation skills are correlational only. Second, some variables previously identified as associated with translational research ability, such as frequency and level of continuing education and workload, were not available for this study. Finally, the study sample was drawn from registered attendees of a large nutrition conference. There is no way to determine the extent to which survey respondents are similar to or differ from the population of dietitians and other health care workers. This, of course, limits external validity. Nevertheless, the study population is large, which improves response prevalence estimates.

## 5. Conclusions 

Our findings reveal a deficit in knowledge translation in a convenience sample of dietitians and other health professionals, highlighting the need to develop general research and knowledge translation skills. Methods to accomplish this might include continuing education courses in a variety of settings (classrooms, online courses and webinars); workplace continuing education courses; required professional education for clinical license renewal is not currently mandatory in Israel.

## Figures and Tables

**Table 1:** Characteristics of survey participants.

Characteristic	Result
Age (years)	38.8 ± 10.9
Sex (% female)	93.3
Profession (%)	
Dietitian	79.0
Student/Intern	8.0
Health Promotion	4.7
Food Industry	2.3
Nurse	2.0
Physician	1.3
Researcher	1.3
Other	1.3
Highest Level of Education (%)	
BSc, BA	57.3
MSc, MA, MPH, MBA	37.5
PhD	3.8
MD	1.4
Employment Setting (%) *	
Private Clinic	34.1
Hospital	28.8
Health Maintenance Organization Clinic	26.7
Long Term Care Facility	10.0
Food Industry	6.0
Other	25.1
Research Participation (%)	20.7
Research Papers Read Monthly (%)	
0–2	41.5
3–5	29.4
6 or more	29.1

* Where the employed provided the option of multiple answers; 60.7% indicated a single location of employment; however, 28.6% of respondents indicated 2 locations of employment, and 10.7% of respondents indicated 3 locations of employment. BSc—Bachelor of Science; BA—Bachelor of Arts; MSc—Master of Science; MA—Master of Arts; MPH—Master of Public Health; MBA—Master of Business Administration; PhD—Doctor of Philosophy; MD—Medical Doctor.

**Table 2:** Response to questions.

Vignette/Response Set (% Per Answer)	Response (%)
Vignette 1: A study was published in which human blood cells exposed to aspartame underwent dysregulated division typical of cancer. Based on this information, what would you advise patients/clients?	(A) I would advise clients to exercise caution in consuming aspartame (29.8%) (B) I would recommend that clients limit their intake of aspartame (38.1%) (C) I would recommend that clients refrain from consuming aspartame (5.7%) (D) I would not make recommendations based on such a study (26.4%)
Vignette 2: A patient with a rare and clinically complex kidney disease is referred to you for treatment, and it is the first time you’ve encountered this condition. What information sources would you consult in order to determine the best nutrition therapy for this patient? Check all that apply.	(A) I would look for formal treatment guidelines (86.6%) (B) I would read studies reporting on new treatments using animal models of the disease (25%) (C) I would employ the most conservative treatment I know of for patients with kidney disease (22%) (D) I would consult with a physician (65.2%)
Knowledge question: When an individual loses weight, where does the excess fat go?	(A) The fat is transformed into energy/heat (2.7%) (B) The fat is excreted as water in urine/sweat (66.6%) (C) The fat is converted to muscle (1%) (D)The fat is expelled as CO_2_ (14.4%) (E) The fat is excreted in the stool (1.3%) (F) I don’t know (9%)
